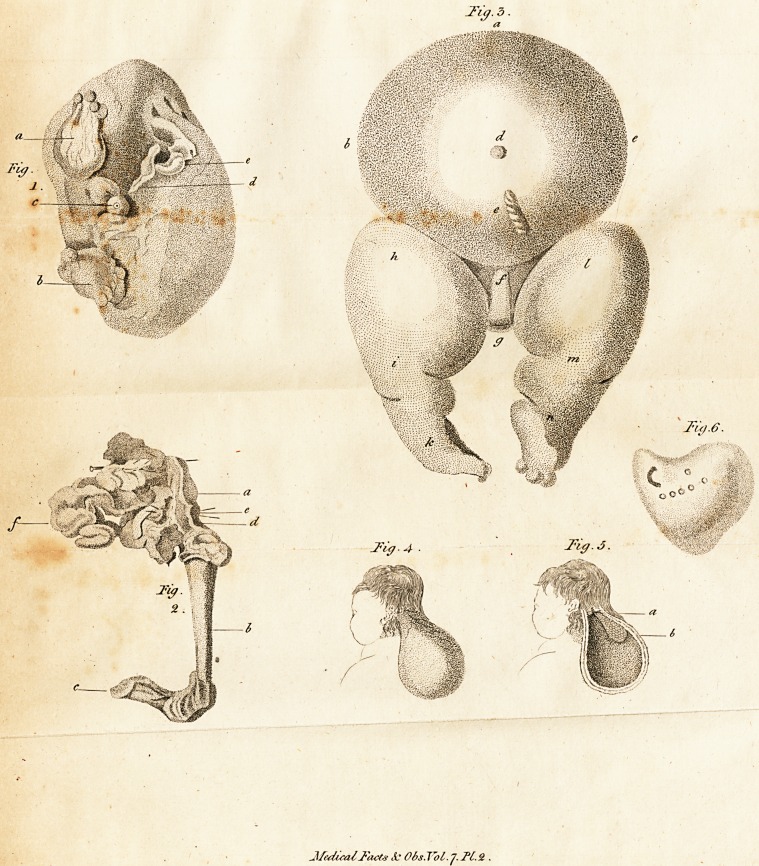# Description of an Extraordinary Production of Human Generation; with Observations

**Published:** 1797

**Authors:** John Clarke


XII. *Description of an extraordinary Production of Human Generation; with Observations. By* John Clarke, *M. D.* Vide *Philosophical Transactions of the Royal Society of London for the Year* 1793. Part II. 4to. London, 1793.

THE expulsion of the monstrous production here described succeeded the birth of a perfect and healthy child, in the General Lying-in Hospital in London. It was inclosed in a distinct bag of membranes, composed of decidua, chorion, and amnios, and had a placenta belonging to it, the side of which was attached to the placenta of the perfect child.

The substance contained in the membranes was covered with the common integuments, and of an oval figure, about four inches in length, and three in breadth; and near the centre of it there was a small funis, about an inch and a half in length, by means of which it was connected to the placenta.

On its surface were seen two imperfect resemblances of feet, on one of which were one l large large and three ſmaller toes, and on the other one large and two ſmaller toes.

Between the two feet was ſituated a ſmall and rounded projection, into which a ſmall paſſage led, capable of containing a briſtle, but it ſoon terminated in a *cul de ſac*. Cloſe to the funis there was another ſmall and thin projection, about a third of an inch in length, which looked like a finger, and was found to contain bony matter, and joints.

There was no appearance of head or neck; of clavicle, ſcapula, or upper extremities; of legs or thighs; or of organs of generation. The only external marks of reſemblance it had to a human fœtus conſiſted of its covering, and the attempt at a formation of two feet and a finger.

Before its internal ſtructure was examined, the navel ſtring of the perfect fœtus was injected; and from this, we are told, the injection very readily paſſed through both placentæ, and even into the ſubſtance of this monſtrous production, as appeared by the redneſs of the ſkin.

On diſſecting off the ſkin it appeared, that of the two imperfect feet, the upper one was connected to the internal parts only by cellular membrane; membrane; but that the lower one was articulated to the inferior parts of a tibia and a fibula.

Internally this production was found to consist of an homogeneous fleshy texture, (very vascular, but without any regular or distinct arrangement of muscular fibres) surrounding an os innominatum, os femoris, tibia, and fibula. Of these bones the two first, it seems, were perfect, and of the size usually met with in a fœtus at the full period of uterine gestation; but the tibia and fibula were much shorter than in their natural proportion to the thigh bone.

At the upper part, and towards the inside of the os innominatum, was found a little portion of small intestines, loosely connected, by their mesentery, to the posterior edge of that bone, where it is commonly united to the os sacrum. These intestines, it seems, had a covering of peritonæum, and were very minutely injected.

Our author's next object was to trace the vessels of the funis. There appeared to be only two, viz. an artery, and a vein; and these passed on towards the inner surface of the os innominatum. As they approached this bone, they gave off some branches to the surrounding parts, which quickly became too small to be traced. The trunks then paſſed backward, towards that part where the articulation with the os ſacrum is generally found; at which place they went to the other ſide of the bone, where they diſtributed a great number of ſmall branches, and were at length loſt in the ſurrounding parts.

This, we are told, was the whole of the internal conſtruction of this very extraordinary monſter. There was not the ſmalleſt appearance of vertebræ, or ribs. There was neither brain, ſpinal marrow, nor nerves. It had no heart, nor lungs. It contained none of the viſcera ſubſervient to digeſtion, excepting the inteſtines already mentioned; nor any glandular ſubſtance whatſoever.

To his deſcription of this ſingular production, which is illuſtrated by two very accurate engravings *, Dr. Clarke has added ſeveral judicious

* In the annexed plate theſe are copied on a reduced ſcale of one half the diameter of the original engravings. See Fig. 1. and 2. of Plate II. Fig. I. exhibits a view of the external appearances: in this figure *a* refers to an imperfect formation of a foot, with four toes upon it; *b* to an imperfect formation of another foot, connected to the tibia and fibula, and having three toes upon it; *c* to the projection into which

Fig. 1.

Fig. 3.

Fig. 6.

Fig. 2.

Fig. 4.

Fig. 5.

dicious and interefting obfervations, which the circumftances of the cafe feemed to him naturally to fuggeft.

The mere defcription of any monfter, as he very properly remarks, is of little utility, unlefs it tends to explain fome actions of the animal economy, before imperfectly, or not at all underftood. It is on this account, he obferves, that very few additions have been made to the ftock of our knowledge, from confidering thofe monfters in which there are either fupernumerary or confufed parts; becaufe, if we cannot which a duct led, terminating in a blind pouch; *d* to the funis umbilicalis; and *e* to an imperfect formation of a finger. In Fig. 2. which exhibits an internal view of the parts, as they appeared after clearing away the flefhy matter from the bones; *a* refers to the os innominatum; *b* to the os femoris; *c* to the tibia and fibula, to which the lower foot was connected; *d* to the funis umbilicalis; *e* to two briftles paffing, in the veffels of the funis, to the outfide of the os innominatum; and *f* to a portion of fmall inteftines, terminating in a *cul de fac* at each extremity.

In this figure, through a miftake of the engraver who copied it, the line of reference at *d* is made to terminate at the outfide of the os innominatum, inftead of extending (as at *d*, fig. 1.) to the funis umbilicalis; the fituation of which, however, is fufficiently pointed out by the inner ends of the two briftles referred to at *e*.

 diftinctly diſtinctly perceive the uſe, or neceſſity of parts, in their natural ſtate, we are not likely to advance in information by the examination of thoſe varieties of ſtructure, where difficulties are only multiplied by the greater complication, or aggravated by the confuſion of parts. The only uſeful inference in natural hiſtory which, he thinks, can be drawn from monſters of the laſt kind is, that nature can deviate from the uſual arrangement of parts, without any material inconvenience; and therefore, that the exiſtence of parts ſo as to be capable of being applied to the purpoſe for which they are intended, in the perfect ſtate of the ſyſtem, rather than any preciſe order of them, is required for carrying on the functions of an animal body.

Monſters, however, in the ſtructure of which conſiderable parts are wanting, ſeem, to him, peculiarly likely to aſſiſt us in the proſecution of phyſiological reſearches. For if we were never to ſee an animal except in its perfect ſtate, we could, he obſerves, form no juſt idea of the comparative neceſſity of the different parts; and if we were to attend only to the complete ſtructure which obtains in the more perfect animals, we might be led falſely to conclude, conclude, that the ufual connexion of parts, which we find in them, is effential to the ftructure and compofition of animal matter. Of thefe parts, the brain and nerves, the ftomach and digeftive organs, the heart and lungs, appear to be of fuch importance, that one might be induced to imagine that the functions of life could not be carried on without them: but in tracing the works of nature downwards, fays our author, we fhall at length find animals gradually becoming more and more fimple in their conftruction. The brain and nervous fyftem are altogether wanting in fome, and there are others which have neither heart nor lungs; yet they continue to exift, and are capable of performing the moft important functions of animals: and thus, he adds, the formation of one animal ferves to throw light upon the economy of others.

Dr. Clarke is aware that this great fimplicity of ftructure is found chiefly in animals, the texture of whofe bodies is nearly homogeneous; not confifting, as in more perfect animals, of parts fo different from each other, as fkin, inteftines, &c. are from bone: and that it may therefore ftill be fuppofed, that all the complicated mechanifm, found in the more perfect animals, is effential to the conftruction of fuch heterogeneous fubftances as thofe of which they confift.

To inveftigate this matter, he thinks, we muft have recourfe to thofe monfters in which there is a deficiency of parts.

There is, he obferves, a very material difference between the nature of the life of the more perfect animals, during their fœtal exiftence, and after their birth. In the latter ftate, the brain and nerves appear to be fo effential, that any very confiderable defect in them is incompatible with the well-being of the animal; but in the fœtal ftate, confiderable deviations from the ordinary arrangement of parts, and fuch as cannot be endured after birth, are fupported without any inconvenience.

In proof of this our author remarks, that the brain has been frequently found very incompletely formed, and fometimes not at all, yet ftill there have been nerves; and that in other cafes, where the brain has been perfect, the fpinal marrow has been deficient in a great part of its extent, and fometimes throughout.

Both thefe circumftances, he thinks, are

ſufficient to prove, that, at any rate, that intimate connexion of the brain and nervous ſyſtem, which takes place after birth, is not neceſſary for the formation of a body in other reſpects perfect. But ſtill, he adds, it would remain doubtful, whether any regular ſtructure could be formed, without any veſtige of either brain or nerves; and therefore without a poſſibility of their influence, in any manner, toward ſuch ſtructure.

The monſter, which is the ſubject of this paper, is, he obſerves, ſo extremely ſimple, in this reſpect, that it cannot be exceeded by the moſt ſimple animal known.

To thoſe who may be diſpoſed to object that there might be brain, or nervous fibres, in this monſter, but that they might, in the diſſection, be deſtroyed, our author replies, by obſerving, that the parts were examined too carefully to warrant ſuch a ſuſpicion; and that as there were no bones repreſenting either the cranium, or ſpine, or os ſacrum, it is not probable that their contents ſhould exiſt in any other ſituation.

He is aware that another objection may perhaps be taken from the anaſtomoſis of the veſſels of the monſter, with thoſe of the perfect fœtus, and that the nervous influence may be fuppofed to have been tranfmitted, in this way, along the veffels; but he contends, there is very good reafon for believing that the veffels of the placenta have no nerves; and that even if they had, it is ftill very unlikely that, merely by fuch anaftomofis, any nervous influence could be conveyed.

Dr. Clarke has thought it right to anfwer another objection which may be made, viz. that nervous matter may be co-extended, or co-exiftent with all other animal matter, and that, of courfe, it is of no confequence whether there be any fenforium, or refervoir of impreffions, &c. or not; becaufe the ftimulus, which produces action, muft refide in parts, as well as the other fubftance of which they are compofed: but although this may poffibly be true, we have, he contends, no evidence of the fact fufficiently fatisfactory to carry conviction along with it. On the contrary, he thinks there is good reafon for believing that nervous influence is conveyed from the brain downwards; and if we are right in this conjecture, which is warranted by the experiment of tying, or cutting nerves, then, he obferves, the exiftence of the nervous fibre, like that of a ftring of a mufical inftrument, would be inactive, unlefs it received ceived an impreſſion, which, with regard to the nerves, ſhould come from the brain.

The whole, then, of the actions of this monſter muſt, he thinks, have been thoſe of the vaſcular ſyſtem entirely; and theſe, he obſerves, ſeem to have been capable of forming bone, ſkin, cellular ſubſtance, ligament, cartilage, inteſtines, &c.

From the defect of heart in this monſter, Dr. Clarke argues, that the energy of the arteries was equal to carrying on the circulation, not only in its own body, but alſo through its own placenta; and from the deficiency of its nerves, that the uſe of theſe is probably very ſmall, if any, to the fœtus.

He mentions it as an opinion entertained by a very acute phyſiologiſt, the late Mr. John Hunter, that in all caſes a fœtus is a very ſimple animal, as to its internal actions; and the circumſtances attending this monſter, he thinks, fully confirm ſuch an idea.

Dr. Clarke obſerves, that in the formation of a fœtus the uſual objects of nature ſeem to be that it ſhould grow, and that it ſhould be fitted with parts which, though of no uſe to it then, are eſſential afterwards. We know, ſays he, that the lungs are of this kind, and it is very likely, he adds, that the brain and nerves are ſo too. That there is a very material difference between the internal functions of a fœtus in the womb, and thoſe of an infant after birth, ſeems, he thinks, very preſumable; not only from our finding that it can carry on life without parts which are of the greateſt moment afterwards; but alſo from its poſſeſſing parts which after birth go into decay, or diſappear, as the thymus gland, &c.

The common uſes of the nervous powers are, to convey impreſſions from without, and volition from within; but a fœtus in the uterus, obſerves our author, is expoſed to no external impreſſions, and is moſt probably incapable of volition, ſince it is not conformable to the general wiſdom of nature to give that which, in ſuch a ſituation, muſt be uſeleſs. He ſeems, therefore, inclined to think that the formation and growth of a fœtal body depend entirely on the actions of its vaſcular apparatus.

## Figures and Tables

**Fig. 1. Fig. 2. Fig. 3. Fig. 4. Fig. 5. Fig. 6. f1:**